# The Effects of 6 Weeks of *Tribulus terrestris* L. Supplementation on Body Composition, Hormonal Response, Perceived Exertion, and CrossFit^®^ Performance: A Randomized, Single-Blind, Placebo-Controlled Study

**DOI:** 10.3390/nu13113969

**Published:** 2021-11-07

**Authors:** Diego Fernández-Lázaro, Juan Mielgo-Ayuso, Miguel del Valle Soto, David P. Adams, Jerónimo J. González-Bernal, Jesús Seco-Calvo

**Affiliations:** 1 Department of Cellular Biology, Histology and Pharmacology, Faculty of Health Sciences, Campus of Soria, University of Valladolid, 42003 Soria, Spain; 2 Neurobiology Research Group, Faculty of Medicine, University of Valladolid, 47005 Valladolid, Spain; 3 Department of Health Sciences, Faculty of Health Sciences, University of Burgos, 09001 Burgos, Spain; jejavier@ubu.es; 4 Department of Morphology and Cell Biology, University of Oviedo, 33003 Oviedo, Spain; miva@uniovi.es; 5 Health Research Institute of the Principality of Asturias (ISPA), 33011 Oviedo, Spain; 6 Dual Enrollment Program, Point University, Savannah, GA 31419, USA; David.Adams@point.edu; 7 Physiotherapy Department, Institute of Biomedicine (IBIOMED), Campus de Vegazana, University of Leon, 24071 Leon, Spain; dr.seco.jesus@gmail.com; 8 Visiting Researcher of Basque Country University, Psychology Department, Faculty of Medicine, 48900 Leioa, Spain

**Keywords:** *Tribulus terrestris*, CrossFit, ergogenic aid, sport performance, hormonal response, body composition

## Abstract

*Tribulus terrestris* L. (*TT*) supplementation have been shown to enhance sports performance in many but not all studies. Moreover, data regarding the potential impact of *TT* supplementation on CrossFit^®^ endurance is limited. This study aimed to determine whether *TT* supplementation improve body composition, hormonal response, and performance among CrossFit^®^ athletes. In a randomized, single-blind, placebo-controlled trial, a total of 30 healthy CrossFit^®^-trained males were randomly allocated to receive either 770 mg of *TT* supplementation or a placebo daily for 6 weeks. Body mass, fat mass, fat composition, testosterone and cortisol levels, and CrossFit^®^ performance (5 common Workouts of the Day: back squat, bench press, dead lift, Grace, and CrossFit^®^ Total) were assessed before and after intervention. There were no significant group x time interactions for the outcomes of the study except for testosterone levels and bench press performance (*p* < 0.05). *TT* supplementation did not impact enhance performance or body composition in CrossFit^®^ male athletes. However, *TT* supplementation may act as a testosterone booster helping the recovery after physical loads and mitigating fatigue.

## 1. Introduction

Dietary supplements are substances that can be used to add nutrients to one’s diet and that have a potential nutritional or physiological effect [1]. In 2020, global trade in dietary supplements was valued at $140.3 billion, and the forecast for the next decade is estimated at an annual growth rate of 8.6% [2]. Vitamins dominated the market and accounted for 31.4% share in 2020. Botanicals had the second largest share in 2020 and are expected to experience significant growth over the next five years [2]. This growing demand for dietary supplements is because the COVID-19 pandemic has made health status the primary concern of the world’s population [1]. Approximately 50–80% of athletes regularly consume dietary supplements [3,4,5,6]. Nutritional supplements for athletes are intended for a specific population group with special nutritional needs that are established by the intensity and duration of exercise, sport discipline, time of the season, age, and gender [7].

There is a kind of dietary supplements for exercise and athletic performance: herbal supplements [8]. One of them is an extract of an exotic plant, named *Tribulus terrestris* L. (TT), originally from India. TT is a famous traditional Chinese medicine that has been widely used in many countries for thousands of years [9]. The genus Tribulus, a plant component of the family Zygophyllaceae, comprises around 20 species worldwide. Among them is TT, which is composed of saponins, flavonoids, glycosides, alkaloids, and tannins [10]. Steroidal compounds, such as saponins, are heterosides consisting of a glycoside part and a genin, which can be steroidal or triterpene in nature. Steroid genins are derived from a hexacyclic skeleton of 27 carbon atoms, which is the spirostane nucleus [10,11]. Spirostanol and furostanol saponins are considered the most characteristic chemicals in TT. To date, kinds of steroidal saponins have been isolated from TT. Among them, there are 58 kinds of spirostane saponins and 50 kinds of furostane saponins [12]. The steroidal saponins, such as protodioscin and protogracillin, are thought to confer to TT unique biological activities. Saponins are responsible for the positive physiological effects of TT on sexual performance, coronary heart disease, avoidance of ischemia/reperfusion injury to the heart, and modulation of hypertension [10,11]. Moreover, TT could reduce inflammation [13] by attenuating muscle damage [14] and oxidative damage [15]. 

CrossFit^®^ is a multimodal workout system that involves standardized routines [16]. It is used to optimize physical fitness through skeletal muscle strength training, flexibility, power, speed of execution, coordination, agility, and precision [17]. The activity is performed based on functional movements known as “Workouts of the Day” (WODs) [18]. In CrossFit^®^ training time, all WODs are completed at maximum intensity in a quick, repetitive manner with little or no recovery time in between [17]. Thus, CrossFit^®^ extreme conditioning programs are employed involving the training of various energy systems, leading to a perceived “very hard” effort that may be causing detrimental effects on athletes [17]. As a consequence of the heavy physical demands of CrossFit^®^, some studies have shown the nutritional recommendations are insufficient, so the athlete may resort to supplements to improve his or her sports performance [19,20]. Thus, nutritional aids help to meet the CrossFit^®^ athletes’ specific nutritional needs, maintain good health, and improve and maximize their athletic performance [19]. 

Currently, products with TT extracts are commonly used by many athletes of different sports modalities [11,21]. Because the continuous intense exercise directly influences an inadequate functioning of the hypothalamus-pituitary-testes axis [22], this alteration may suppress testosterone secretion in the late stages of physical activity, which would cause performance to be compromised [22,23]. Athletes could supplement with nutritional aids with potential androgenic effects to avoid the use of steroids for two main reasons: (1) in order to not suffer side effects and (2) not to test positive in an anti-doping control [24]. TT is attractive for athletes because of its potential ergogenic effects on sports performance [25], facilitating recovery after exercise [26], improvement of strength and stimulation of skeletal muscle hypertrophy [27] associated with TT as a testosterone booster [21], and promoter of muscle anabolism [11,26]. Nonetheless, more clinical trials are needed to obtain honest evidence on the efficacy of TT.

To our knowledge, no research has examined the impact of TT supplementation on CrossFit^®^ performance. Potentially, WODs could be a suitable model for determining the effects of TT on CrossFit^®^-trained men by employing high volumes and intensities of training in various modalities and durations. Consequently, it is considered a novel exercise intervention that tests body systems in a balanced and integrated manner, challenging people’s abilities to complete mechanical work. As such, we aim to evaluate high-intensity training regimens of CrossFit^®^ athletes with TT supplementation on WODs sports performance, hormonal response as regarding testosterone and cortisol, body composition, and perceived exertion produced by the CrossFit^®^ training program under these conditions.

## 2. Materials and Methods

### 2.1. Design and Participants

A randomized, single-blind, placebo-controlled trial was conducted to evaluate the effects of TT supplementation over 6 weeks on the sport performance and hormonal response of CrossFit^®^-trained athletes. A break of a couple of weeks is recommended every 6–8 weeks of supplementation with TT (laboratory duration recommendation). Additionally, it is not recommended to consume TT for more than 6–8 weeks in a row without a break as an androgenic enhancer [28] or anabolic precursor of serum testosterone concentrations [29] in humans. Toxicity studies in animals had a maximum duration of 8 weeks [30]. Inclusion criteria included at least 20 months of CrossFit^®^ experience, previous completion of “Fran” (WODs) (three rounds of thrusters and pull-ups for 21, 15, and 9 repetitions) in less than 250 s, males between 18 and 50 years of age, and a signed informed consent document. The participants were studied using a cardio-pulmonary and electrocardiographic examination and required to complete a medical questionnaire prior to entry into the study. Exclusion criteria included any preexisting physical health issues, consuming alcohol, or taking either medications or illegal substances capable of altering muscular, hormonal, or athletic performance response. There were no injuries before or during the performance of the test, as they were ruled out by history and clinical examination. All CrossFit^®^ athletes followed the same diet during the study, supervised by a qualified nutritionist.

Thirty male CrossFit^®^-trained athletes volunteered for the study, authorized by the Clinical Research Ethics Committee (CREC) and Committee on Drug Research Ethics (CDRE) of Valladolid East Area (PI-19-1350), from two gyms in Soria and Salamanca, Spain. Bioelectrical impedance (BC-730; Tanita, Japan) was used to assess body mass and fat mass. Resistance and reactance measurements were obtained with a bioimpedance analyzer (BC-730; Tanita, Japan), by a single observer, with a constant alternating current of 800 µA and frequency of 50 kHz. A tetrapolar configuration was used on hands and feet on the right side of the body, with disposable electrodes. The distal electrodes or signal are placed on the dorsal surface of the hands and feet, proximal to the metacarpal and metatarsophalangeal joints, and the sensors are located below an imaginary line passing through the ulnar and radial facet joints and the intermalleolar line. Measurements were performed in the supine decubitus position, with a separation between the legs of 45° and the arms 30° with respect to the trunk. A minimum fasting period of 4 h was required, urinating 30 min before, and not having done any physical exercise in the previous 24 h, avoiding coffee and any type of diuretic. The temperature of the room where the measurements were taken was controlled to ensure a neutral environment. Resistance and reactance readings were collected in triplicate using their mean for the calculations. The calculation of free fat mass was performed with an equation Free Fat mass = 0.82 × size^2^/R + 0.86. The free fat mass included muscle, organs, bone, and body water [31]; a stadiometer was used to measure height. Two TT capsules from the Quamtrax laboratory (Quamtrax Nutrition Europe S.L. Fuenlabrada, Madrid, Spain) were administered as a single dose of 770 mg daily on an empty stomach. TT extract was 72.64% (385 mg/cap) (found in the active principles), with 40% of saponins and 27.36% residue, with other ingredients including gelatin (empty capsule), zinc sulfate 5.18% (27.47 mg/cap), maltodextrin, anti-caking agent (magnesium stearate), and color E-171 titanium dioxide (empty capsule). Two 100-mg maltodextrin capsules were used as a placebo to match the color and texture of the TT tablets to ensure blinding. The placebo was performed in the laboratories of the Magistral Formulation Laboratory of a Pharmacy (Soria, Spain).

A member of the research team was responsible for instructing the subjects on how to take the supplement, reminding them how to take it (in person and/or by phone), and providing them with strategies to try to achieve 100% adherence (pill dispensers and calendars).

The randomization sequence was generated by a statistician, which allowed the athletes to be assigned to the two study groups using a stratified block design: control group (CG) and intervention group (IG) treated with 770 mg day^−1^ of TT (Table 1).

### 2.2. Hormone Determination in Peripheral Blood

Total testosterone was determined by enzyme-linked immunosorbent assay (ELISA) (DRG Instruments GmbH, Marburg, Germany). Cortisol concentration was assessed by enzyme-linked fluorescence assay (ELFA)-based technology by ready-to-use reagents with a compact multiparametric immunoanalyzer Minividas^®^ (Biomerieux, Marcy l’Etoile, France). Percent variations in plasma volume (% ΔPV) were estimated using Van Beaumont’s method. Moreover, analytical marker values were adjusted using the following equation: Corrected value = Uncorrected value × [(100 + % ΔPV)/100)] [32,33]. 

### 2.3. CrossFit Training

During the 6 weeks of the study, training involved 3 weekly sessions on alternate days (Monday, Wednesday, and Friday). Each session was one-hour long and was divided into a CrossFit^®^ warm-up, strength and/or CrossFit^®^ skill technique as well as a structured strength or conditioning workout between 10 to 30 minutes, followed by stretching and/or cool down. Each workout was performed in the participants’ own gym, supervised by a certified CrossFit^®^ Degree 1- or 2-certified instructor (at each). All subjects completed the same physical activity routines to ensure that they performed the same prescribed workout during the study.

### 2.4. Dietary Evaluation

A Registered Professional Dietician Nutritionist carefully documented in detail the subjects’ daily food and fluid intake throughout the trial. The CrossFit^®^ athletes were briefed on two diet-tracking systems [32,33]: (i) a food frequency questionnaire (FFQ) at T2 and (ii) a questionnaire dietary recall of the previous 7 days to T2. This 7-day dietary recall questionnaire was performed to verify that the results were like those obtained with the FFQ. Values for each food were transformed into micronutrients, macronutrients, and total energy intake. The validated Easy Diet© software was used for this purpose. In addition, the total energy intake/kg was calculated for each athlete. 

### 2.5. Evaluation of Sports Performance

The performance of subjects was evaluated through different WODs following the international CrossFit^®^ protocols [16,18]. The tests performed were the back squat, bench press and deadlift, CrossFit^®^ Total, and Grace. For CrossFit^®^ Total, athletes must complete under supervision one repetition maximum (RM) of bench press, squat, and deadlift in a time of 90 min. For each lift, beginning with the back squat WOD, a progressive individual warm-up was performed, starting with a lift of around 50% of a RM for 10 min. After the precise warm-up for each lift, subjects were authorized three attempts to achieve a one RM, with 180–300 s of rest between each. This methodology was also utilized for the dead lift and bench press. In kilograms (kg), the individual loads of an RM were summed to determine the total score. At Grace, 30 repetitions of clean and jerks were performed, and the time in seconds (s) to complete them was measured. Male athletes were obliged to employ 61.4 kg weights [16,18]. In the first movement, the clean lift, the competitor lifts the bar from the ground by performing a jerk and squat to get under it. In the second movement, the jerk lift, by minimally bending the knees, he lifts the bar above his head, taking momentum from his legs and extending his arms entirely. The lifter should keep the feet in the same plane throughout the process and stretch the legs fully in the second phase [16,18].

### 2.6. Determination of Perceived Exertion

CrossFit^®^ athletes were monitored in baseline (T1) and post-treatment (T2) of rated perceived exertion (RPE) using the Borg CR-10 scale. RPE runs from 0–10 to measure how easy or difficult one finds a workout [32]. Before blood collection at each time point, participants were asked to rate their perceived muscle discomfort at each one (T1 and T2) using this scale. At the beginning of the study, all subjects were instructed to utilize the Borg CR-10 scale to assess their perceived muscle discomfort throughout the study. Adequate validity and reliability of this tool have been reported following the procedure described. Because the scale was being used to rate “discomfort” rather than “pain,” participants were informed that “maximum pain” should be used as a starting point equating to their worst local muscle discomfort previously experienced during physical activity and that all other ratings should be related to this [32]. 

### 2.7. Blinding

The 30 athletes in the study who received TT supplementation completed a questionnaire to evaluate the blinding procedure. Twenty-seven subjects (90%) were unable to determine to which study group they were assigned. Only 1 of the 3 athletes who indicated that they had an idea of which group they belonged to was correct. This would suggest that the single-blinding process was successful.

### 2.8. Statistical Analysis

The treatment was randomly assigned utilizing a sequence generated by date (or day) of admission in the study by Random Sequence Generator. Random Sequence Generator is an application, which was accessed through the Internet. The data were presented as means and standard deviations. Analyses was performed using SPSS version 4.0 software (SPSS, Inc., Chicago, IL, USA). Statistical significance was indicated when *p* < 0.05. Paired *t*-tests assessed differences between T1 to T2 in each group after the normality of the data was established with the Shapiro–Wilk test (*n* < 50) based on parametric or nonparametric data. A two-way repeated-measures analysis of variance (ANOVA) test was used to examine interaction effects (time supplementation group) among supplementation groups (CG and IG) for body composition, sports performance, and hormonal response. A Bonferroni post-hoc test was applied for pairwise comparisons among groups. The percentage changes of the variables studied in each study group between T1 and T2 tests were calculated as ∆ (%): [(T2 − T1)/T1 × 100)] for CG and IG. Effect size among participants were calculated using eta-squared (η2p). The η2p is a measure of effector size association. Because this measure often overestimates effect size, values were interpreted as the following: where no effect is indicated, if 0 ≤ η2p < 0.05; a minimal effect is shown if 0.05 ≤ η2p < 0.26; a moderate effect is indicated if 0.26 ≤ η2p < 0.64; and a strong effect is indicated if η2p ≥ 0.64. 

## 3. Results 

### 3.1. Dietary Evaluation

There was no significant difference between groups (CG and IG) (*p* > 0.05) for energy, proteins, fats, carbohydrates, micronutrients, and vitamins (Table 2).

### 3.2. Body Composition

In Table 3, the body mass, fat mass, and free fat mass of the CG and IG at T1 and T2 are shown. In both groups, all body composition parameters showed significant differences during the study (*p* < 0.05). However, the group-by-time interaction between CG and IG did not reveal significant differences (*p* > 0.05).

### 3.3. Workouts of the Day (WODs), Hormonal Response, and Rate Perceived Exertion (RPE)

Table 4 shows the results of the WODs (CrossFit^®^ Total and Grace) for both GC and IG at the beginning and end of the study (T1 and T2, respectively). Within the CrossFit^®^ Total tests (bench press, squat, and deadlift), only a significant increase (*p* < 0.05) was observed in the bench press in GI (T1: 69.17 ± 11.58 vs. T2: 80.00 ± 11.40). In addition, significant differences (*p* < 0.05) were only observed in group-time for the bench press (*p* < 0.05; η2p = 0.346). In both groups, a shorter execution time was observed, without statistical significance (*p* > 0.05), in the Grace test in both GI (T1: 28.00 ± 6.23 22.67 ± 5.92 vs. T2: 22.67 ± 5.92 28.00 ± 6.23) and CG (T1:30.17 ± 5.08 vs. T2: 24.33 ± 4.46). No significant group-time differences were observed for the Grace test (*p* > 0.05; η2p = 0.017).

In addition, Table 4 shows that in both groups, CG and IG, no significant differences (*p* > 0.05) were observed between baseline and at the end of the six-week study in testosterone, cortisol, and testosterone/cortisol ratio. Nor were there significant differences in group-by-time for cortisol (*p* > 0.05; η^2^p = 0.211) and ratio testosterone/cortisol ratio (*p* > 0.05; η^2^p = 0.228). Significant differences (*p* < 0.05) can be seen in the group-by-time for testosterone (*p* < 0.05; η^2^p = 0.495).

Finally, Table 4 displays the RPE in the CG and IG, and the Borg CR10 scale indicates a non-significant increase (*p* > 0.05) in the perceived fatigue between T1 vs. T2 in both groups. Additionally, there were no significant differences in the group-by-time for RPE (*p* > 0.05; η^2^p = 0.186).

Table 5 shows the percentage changes on WODs between groups and significant differences (*p* < 0.05) in the bench press test between the IG vs. CG. There were no significant differences (*p* > 0.05) in dead lift (IG 4.64 ± 2.55 vs. CG 3.32 ± 4.17) and CrossFit^®^ Total (IG 6.19 ± 3.19 vs. CG 5.07 ± 4.61), but there was an improving trend in the IG group. However, in the CG the percentage change was greater for squat than IG (CG 5.70 ± 8.303 vs. IG 3.01 ± 4.96). The results for the CrossFit^®^ Total test were similar for both groups (CG −24.73 ± 10.58 vs. IG −25.00 ± 10.74). 

In addition, Table 5 shows the percentage changes during the study on hormonal response; significant differences (*p* < 0.05) were observed in the testosterone levels (IG −0.20 ± 13.63 vs. CG −21.85 ± 11.10). There were no significant differences (*p* > 0.05) in cortisol (IG 4.64 ± 2.55 vs. CG 28.65 ± 43.19) and testosterone/cortisol ratio (IG 22.43 ± 69.60 vs. CG −29.07 ± 37.38), but there was an improving trend in the IG group. 

## 4. Discussion

The purpose of our investigation was to evaluate the impact of six-weeks TT supplementation (770 mg · day^−1^) on body composition, hormonal response, RPE, and CrossFit^®^ performance in CrossFit^®^-trained males. We hypothesized that TT supplementation would improve CrossFit^®^ performance and testosterone levels since previous studies in athletes demonstrated that TT enhanced sports performance [25,34] during intense periods of training, modulated muscle damage from high intense exercise [14,25], and increased plasma testosterone concentration and muscle mass [35]. Our hypothesis was partially supported by significant differences that were observed in bench press test and testosterone levels between IG vs. CG in different phases of the study (T1 vs. T2). However, the results revealed that TT had no significant effect on WODs (CrossFit^®^ Total and Grace test), body composition parameters (body mass, fat mass, free fat mass), cortisol, and testosterone/cortisol ratio.

### 4.1. TT Does Not Alter Body Composition

Significant differences were observed in all measures on body composition over the six weeks regardless of group (IG or CG) and with no significant difference in the group-time interaction, indicating that the CrossFit^®^ training program would be responsible and not the TT supplementation. Perhaps, the dose of TT administered to the IG would not be the optimal dose to achieve notable changes in body composition compared to the CG. Our results are in agreement with other studies in male athletes [25,27,36,37] or in a murine model [38]. However, in a study in weightlifters, the CG compared to the supplemented with TT group had relatively less change in muscle mass after 12 weeks of training [35]. 

These results would suggest that the increase in bench press performance in IG was not mediated by changes in body composition or increased muscle mass. This could be because of the adequate adaptability of the IG athletes to the CrossFit^®^ training program, and/or the absence of TT could have led to a lower level of adaptation in CG. Therefore, the information from bodybuilding and fitness websites on the activity of TT compound supplements in decreasing body fat and increasing lean muscle mass is questionable [9,11]. 

### 4.2. TT on CrossFit^®^ Performance

We did not observe significant improvements in WODs, which evaluates global strength by the sum of the measurement of 1 repetition maximum (RM) of bench press, squat, and dead lift. These results are similar to those reported in a study conducted in rugby players, where no significant improvement in total strength was observed after supplementing for five weeks with 450 mg · day^−1^ of TT [27]. In addition, in resistance-trained men supplemented with TT (3.21 mg · kg^−1^ for 8 weeks) [36], researchers also did not observe significant improvement in total strength. When analyzing each CrossFit^®^ Total exercise, squat and dead lift showed no significant gains. The squat showed a slightly better percentage improvement in IG than in CG. However, in dead lift, the opposite was true. Squat and dead lift employ muscle groups, mainly of the posterior chain, involved in many other CrossFit^®^ movements (snatch, thrusters, lunges, burpees, box jump). This could indicate that habitual training is responsible for the individual adaptations of each athlete [27], which in squat and dead lift, are strength and muscle mass development [16,18]. 

Alternatively, the bench press showed significant improvements in the IG between T1 and T2, twice as much as in the CG. Our results are similar to those obtained in 1-RM bench press after three weeks of TT supplementation in powerlifters [12,21]. The bench press gesture involves muscle groups (pectoralis major, anterior deltoid, triceps brachii, and latissimus dorsi) that are not usually used in other WODs [17,18]. Thus, TT-mediated actions would be responsible for increasing bench press performance, such as stimulating endogenous testosterone [12,21,24]. Other alternatives to the positive effect of testosterone on athletic performance [23] could be the increase in the number of muscle androgen receptor (AR) (AR is testosterone receptor) [12,39], the inhibition of insulin-like growth factor-1 receptor (IGF-1R) (increased by overtraining) [26,39], and the improvement in insulin-like growth factor-1 (IGF-1) bioactivity in plasma by the decrease in the concentration of insulin-like growth factor binding protein 3 (IGFBP-3) [25,39]. 

TT-mediated inhibition of cyclooxygenase-2 (COX-2) [40] could also increase nitric oxide (NO) production. NO is a potential modulator of blood flow activation, increased muscle energy metabolism, and enhanced mitochondrial respiration during exercise. Thus, stimulating the biosynthesis and improving the accessibility of nitric oxide could have positive effects on performance in athletes [40]. Milasius et al. [34] reported significant increases in anaerobic performance after 20 days of supplementation with TT capsules of 1875 mg. Similar results, Ma et al. [25] found significantly increased anaerobic performance in males athletes supplemented with TT (1250 mg) for three weeks. In contrast, our results showed no significant discrepancy of Grace test in CG and IG reported during the study. Grace is a WOD in which the anaerobic physical job predominates for its execution [17,18]. Perhaps the difference between the results could be due to the supplementation dose. Therefore, high doses of TT would be necessary to obtain improvements in anerobic performance [25,39], probably mediated by the effects derived from the increase in NO production [40].

### 4.3. TT Unchanged Rate Perceived Exertion

TT is recommended as an herbal supplement that optimizes and increases mood [11,41], probably by contributing to an increase in testosterone levels [26]. Testosterone favor and increase the production of different neurotransmitters, such as dopamine and acetylcholine. These neurotransmitters are involved in maintaining optimal mood, evoking greater feelings of pleasure, and a higher level of energy and activity [42]. However, our data suggested that TT had no difference observed in RPE, as measured with the Borg CR-10 scale, between IG vs. CG at either of the different study points (T1 vs. T2). Thus, TT supplementation did not affect athletes’ perception of exertion, fatigue, or vigor during exercise. These results follow those reported in endurance athletes supplemented for eight weeks with TT 3.2 mg · kg weight [30]. Additionally, a significant increase in testosterone levels (as in IG) had no effect on mood in elite basketball players [32].

### 4.4. TT on Hormonal Behavior

Continuous, intense exercise directly influences an inadequate functioning of the hypothalamus-pituitary-testes axis [22]. This alteration may suppress testosterone secretion in the late stages of physical activity, which would cause performance to be compromised [22,23]. Athletes could supplement with nutritional aids with potential androgenic effects to avoid the use of steroids for two main reasons: (i) not to suffer side effects and (ii) not to test positive in an anti-doping control [24]. In this sense, TT is a testosterone booster and is a popular nutritional supplement in athletes for enhancing sports performance [21]. In studies with animal models, TT stimulation of testosterone production leads to improved athletic performance, enhances recovery after exercise, and prevents overtraining [26,43,44,45]. However, in human studies, TT does not affect testosterone plasma levels in male boxers [25] and rugby players [27]. Additionally, testosterone had no significant changes after a four-week supplementation protocol with TT (10 or 20 mg · kg^−1^ · day^−1^) in young adults [28]. Similarly, testosterone showed no significant changes after treatment with TT (750 mg · day^−1^) for three months in adults with idiopathic infertility [41]. To our knowledge, our study is the first to indicate a significant discrepancy of testosterone with or without TT extracts during training in humans. These results suggest that TT could be useful in maintaining homeostasis of the hypothalamus-pituitary-testes axis, promoting anabolic state, stimulating post-exercise recovery, and improving athletic performance. However, the results could be influenced by type of exercise, amount of each supplement, and duration of the intervention. Participant characteristics, such as age, gender, ethnicity, body composition, training level, differences in training, nutrition, and health status, may also have influenced the results. It has been reported that of the wide variety of molecules with biological properties of TT, steroidal saponins and flavonoids are considered the most important metabolites with multiple bioactivities [12]. In this way, the steroidal saponins (gitonin, protodioscin, and tribulosaponins A and B) present in TT have an effect on androgen receptors in the brain, causing an underestimation of sex hormone levels, which causes the posterior pituitary gland to secrete more LH and, as a consequence, increased testosterone synthesis in the testes [12,26]. Additionally, protodioscin in particular is believed to increase the conversion of testosterone to dihydrotestosterone, which promotes red blood cell production and muscle development [11]. Other mechanisms by which it is thought that TT may be able to increase testosterone levels are by increasing the levels of dehydroepiandrosterone (DHEA) molecules (precursor of testosterone) and by suppressing aromatase and preventing estrogen synthesis [11], and these may be by the activity of steroidal saponins, such as protodioscin and protogracillin [12].

Cortisol is released from the adrenal cortex in response to stressors linked to exercise and catabolic actions; it is also directly influenced by duration and intensity [23]. The cortisol response to TT supplementation was evaluated by Milasius et al. [34] in endurance athletes. Cortisol concentration in blood of IG after 20 days supplementation with 1.875 mg day^−1^ increased, but this change was not statistically significant [34]. Although no significant changes in cortisol were observed at the end of the six-week supplementation, it tended to decrease in IG and increase in CG, suggesting favorable results for IG in cortisol response. Perhaps, the difference in the results may be influenced by the biological profiling or physiological characteristics of the athletes in each group (maximal intensity interval vs. long distance), the type of training (strength resistance vs. endurance), and level of physical fitness of the athletes (CrossFit^®^ trained vs. athletes belonging to a physical education program). These results could indicate that the possible efficacy of TT on hormonal status would be conditioned in relation to the sport modality and/or the physical condition of the athletes.

Testosterone/cortisol ratio is indicative of the anabolism/catabolism ratio in the body due to their role in protein synthesis and degradation. It can be used as a biological indicator to supervise fatigue and establish optimal training loads for athletes [23]. Testosterone/cortisol ratio did not prove to be significant after the TT supplementation protocol. Nonetheless, IG showed a better trend (*p* > 0.05) in the testosterone/cortisol ratio concerning CG at the end of the study, which could translate into a potential better response to training load and volume and establish a positive trend in the recovery process in athletes. In this way, improvements in muscle recovery after high-intensity endurance exercises by reduction of creatine phosphokinase (CPK) and lactate dehydrogenase (LDH) have been reported after supplementation with 500 mg · day of TT [14]. 

### 4.5. TT Extract Components

Nutritional supplements contaminated with banned substances have been reported [46]. Tainted products could cause unintentional doping among unsuspecting athletes [47]. In our study, we have used a commercial Quamtrax laboratory that assures that the TT supplement is doping-free by certified by Ultra High-Performance Liquid Chromatography (UHPLC) analysis. None of the participants in this research reported adverse effects during the supplementation protocol. Despite this, some studies have reported gastrointestinal issues, such as stomach pain or gastric reflux [48,49]. 

### 4.6. Limitations

The present study has certain limitations. The sample size is small; a larger one would produce more robust results. Moreover, the realization in professional athletes of different sports modalities would also give us the accurate dimension of the potential properties of TT supplementation because the nature of each sport has demands that differ between disciplines, such as aerobic or anaerobic capacity, the type of strength used, the predominant energy systems, and other factors that would justify the individualization of TT testing. Additionally, the design exclusively focuses on males, which may reduce generalizability. Nor were biochemical tests included, especially determination of muscle damage marker levels, such as lactate dehydrogenase, creatine kinase, and myoglobin. Thus, it would be necessary to include such as analysis in future studies.

## 5. Conclusions

The dose 770 mg · day^−1^ of TT used in CrossFit^®^-trained men does not appear to influence body composition, RPE, total strength, and anaerobic performance. However, our results suggest that it could potentially intervene in the recovery process by a tendency, although not significant, to increase serum testosterone and maintain a favorable trend in the testosterone/cortisol ratio, which would mean that it could potentially influence less fatigue and catabolism associated with exercise. 

## 6. Practical Applications

TT supplementation could be recommended for athletes playing sports at levels where the competition rules prevent adequate recovery, such as play-offs in team sports (handball, volleyball, basketball, soccer, field hockey) or tournaments in individual sports (tennis, swimming) and in training stages characterized by high loads and volumes. In addition, since the beginning of the COVID-19 pandemic, more males have been affected as well as a higher rate of intensive care unit admissions and deaths. Perhaps a lower bioavailability of testosterone or a decrease in its receptors could be the cause. In this regard, low total testosterone levels are associated with a hyperinflammatory state in hospitalized men with COVID-19 and represent a risk factor for hospital mortality in COVID-19 patients. Therefore, inadequate total testosterone levels may serve as markers of poor prognosis in COVID-19 [50]. Thus, good hormonal health, especially related to testosterone, is directly related to the stability and strength of the immune system. In this sense, TT could be used as a supplement to maintain optimal testosterone levels. Before supplementation, the physiological effects of the substance and the characteristics of everyone should be considered.

## Figures and Tables

**Table 1:** Characteristics of study participants.

	Control Group (CG)	Intervention Group (IG)	*p*
Sample size (*n*)	15	15	
Age (years)	32.9 ± 6.3	33.1 ± 5.7	0.611
Body mass (kg)	81.2 ± 11.5	80.1 ± 10.7	0.354
Height (cm)	174.5 ± 3.3	175.1 ± 2.7	0.871
Crossfit^®^ experience (months)	41.3 ± 17.5	42.4 ± 18.32	0.651
Fran WODs (seconds)	233 ± 12	229 ± 14	0.309

Data are expressed as mean standard deviation. *p*, differences between groups using one-way ANOVA. Workouts of the Day, WODs.

**Table 2:** Energy and micronutrients intake in control group (CG) and intervention group (IG) during 6 weeks of study.

Group	CG *n* = 15	IG *n* = 15	*p*
Energy (kcal/kg)	38.3 ± 5.8	39.7 ± 5.2	0.273
Proteins (g)	145.3 ± 36.9	138.3 ± 44.9	0.395
Fats (g)	139.3 ± 40.2	141.3 ± 42.6	0.748
Carbohydrates (g)	340.2 ± 98.6	345.6 ± 103.2	0.435
Ca (mg)	1036.3 ± 214.1	1082.4 ± 193.6	0.345
Mg (mg)	542.3 ± 99.2	551.1 ± 95.9	0.863
P (mg)	2123.6 ± 66.1	2076.9 ± 84.3	0.583
Fe (mg)	21.1 ± 4.6	23.5 ± 5.7	0.801
Zn (mg)	13.7 ± 0.8	14.7 ± 0.8	0.699
Vitamin A (µg)	1859.3 ± 1180.1	2002.1 ± 775.2	0.659
Vitamin E (mg)	17.0 ± 2.5	17.3 ± 1.6	0.466
Vitamin B_1_ (mg)	2.6 ± 0.2	2.8 ± 0.6	0.526
Vitamin B_2_ (mg)	2.7 ± 0.2	2.7 ± 0.2	0.693
Vitamin B (mg)	40.0 ± 7.1	37.2 ± 3.9	0.815
Vitamin B_6_ (mg)	4.1 ± 0.7	4.3 ± 0.9	0.831
Vitamin B_9_ (mg)	634.2 ± 171.1	636.4 ± 169.5	0.885
Vitamin B_12_ (µg)	9.1 ± 3.9	9.3 ± 3.1	0.877
Vitamin C (µg)	347.1 ± 138.2	356.4 ± 119.6	0.733

Data are expressed as mean ± standard deviation. *p*, significantly different between groups by independent *t*-test. Kilograms, kg; grams, g; milligrams, mg; micrograms, µg; kilocalories, Kcal; control group, CG; intervention group, IG.

**Table 3:** Body composition in the control group (CG) and intervention group (IG) at baseline (T1) and after 6 weeks (T2).

Group	T1	T2	P(TxG)	η^2^p
	Body Mass (kg)			
CG	81.2 ± 11.5	80.7 ± 10.6 *	0.741	0.117
IG	80.9 ± 10.7	79.6 ± 9.3 *		
	Fat Mass (%)			
CG	13.2 ± 5.7	12.5 ± 6.3 *	0.236	0.098
IG	13.7 ± 2.8	12.1 ± 1.8 *		
	Fat Mass (kg)			
CG	10.6 ± 2.1	9.6 ± 1.3 *	0.506	0.063
IG	11.1 ± 1.9	9.3 ± 1.6 *		
	Free Fat Mass (%)			
CG	86.8 ± 5.3	87.5 ± 6.1 *	0.356	0.081
IG	86.3 ± 2.3	87.9 ± 2.3 *		
	Free Fat Mass (kg)			
CG	70.9 ± 2.3	71.1 ± 1.6 *	0.478	0.056
IG	69.8 ± 1.4	70.3 ± 2.3 *		

Data are expressed as mean ± standard deviation. P(TxG), group-by-time interaction (*p* < 0.05, all such occurrences). Two-factor repeated-measures ANOVA. * Significantly different between study points (T1 vs. T2); *p* < 0.05. Control group, CG; intervention group, IG; kilograms, kg; percentage, %.

**Table 4:** Results of the Workouts of the Day (WODs), hormonal response, and rate perceived at baseline (T1) and after 6 weeks (T2).

	Test	Group	T1	T2	P(TxG)	η2p
WODs	Bench Press (kg)	CG	70.83 ± 10.68	76.67 ± 10.80	0.033	0.346
		IG	69.17 ± 11.58	80.00 ± 11.40 *		
	Squat (kg)	CG	142.50 ± 28.94	149.17 ± 23.11	0.496	0.048
		IG	128.33 ± 15.71	131.67 ± 11.25		
	Dead lift (kg)	CG	160.83 ± 19.34	165.83 ± 18.28	0.438	0.061
		IG	162.50 ± 9.87	170.00 ± 10.49		
	CrossFit^®^ Total (kg)	CG	374.17 ± 47.48	391.67 ± 37.24	0.599	0.029
		IG	360.00 ± 5.36	381.67 ± 30.93		
	Grace Test (s)	CG	30.17 ± 5.08	24.33 ± 4.46	0.689	0.017
		IG	28.00 ± 6.23	22.67 ± 5.92		
Hormonal Response	Testosterone (ng/dL)	CG	6.86 ± 2.03	5.34 ± 1.56	0.011	0.495
		IG	5.76 ± 0.86	5.75 ± 1.24		
	Cortisol (ng/dL)	CG	17.10 ± 2.69	22.15 ± 8.81	0.134	0.211
		IG	15.17 ± 3.65	14.30 ± 5.17		
	Ratio T/C	CG	0.41 ± 0.13	0.27 ± 0.10	0.116	0.228
		IG	0.39 ± 0.08	0.49 ± 0.33		
RPE	(Borg CR-10)	CG	7.46 ± 1.23	7.55 ± 1.56	0.91	0.186
		IG	7.48 ± 1.31	7.56 ± 1.47		

Data are expressed as mean ± standard deviation. P(TxG), group-by-time interaction (*p* < 0.05, all such occurrences). Two-factor repeated-measures ANOVA. η2p, the effect of the size.* Significantly different between study points (T1 vs. T2); *p* < 0.05. Control group, CG; intervention group, IG; kilograms, kg; seconds, s; Workouts of the Day, WODs; testosterone, T; cortisol, C; nanograms per deciliter, ng/dL; rate perceived exertion, RPE.

**Table 5:** Percentage changes during study on Workouts of the Day (WODs) in the two study groups: control group (CG) and intervention group (IG).

	Test	CG	IG	*p*
WODs	Bench Press (kg)	8.52 ± 5.54	19.22 ± 7.15	0.044 *
	Squat(kg)	5.70 ± 8.03	3.01 ± 4.96	0.501
	Dead lift (kg)	3.32 ± 4.17	4.64 ± 2.55	0.523
	CrossFit^®^ Total (kg)	5.07 ± 4.61	6.19 ± 3.19	0.635
	Grace Test (s)	−24.73 ± 10.58	−25.00 ± 10.74	0.966
Hormonal Response	Testosterone (ng/dL)	−21.85 ± 11.10	−0.20 ± 13.63	<0.001 *
	Cortisol (ng/dL)	28.65 ± 43.19	−4.64 ± 30.12	0.153
	Ratio T/C	−29.07 ± 37.38	22.43 ± 69.60	0.141

Percentage changes during study. Data are expressed as mean ± standard deviation. Δ, ((T2−T1)/T1) × 100; differences among groups in each test by ANOVA test (*p* < 0.05): * regarding CG. Control group, CG; intervention group, IG; kilograms, kg; seconds, s; Workouts of the Day, WODs; testosterone, T; cortisol, C; nanograms per deciliter, ng/dL.

## Data Availability

Not applicable.
